# Circulating tumor DNA – A potential aid in the management of chordomas

**DOI:** 10.3389/fonc.2022.1016385

**Published:** 2022-10-20

**Authors:** Stephen C. Frederico, Corbin Darling, Xiaoran Zhang, Sakibul Huq, Sameer Agnihotri, Paul A. Gardner, Carl H. Snyderman, Eric W. Wang, Georgios A. Zenonos

**Affiliations:** ^1^ School of Medicine, University of Pittsburgh, Pittsburgh, PA, United States; ^2^ Department of Neurological Surgery, University of Pittsburgh, Pittsburgh, PA, United States; ^3^ Department of Otolaryngology, University of Pittsburgh, Pittsburgh, PA, United States

**Keywords:** clivus, ctDNA, DNA, tumor, skull base, chordoma

## Abstract

Chordomas are a locally invasive, low-grade, CNS malignancy that are primarily found in the skull base, spine, and sacrum. They are thought to be derived from notochordal remnants and remain a significant clinical challenge due to their local invasiveness, resistance to chemoradiation, and difficulty in achieving a complete resection. Adjuvant therapy such as proton beam therapy is critical in preventing recurrence in patients who are at high risk, however this treatment is associated with increased risk of complication. Currently, intraoperative observation and imaging findings are used to determine recurrence and success of gross total resection. These methods can be unreliable due to limited operative view, bony and soft tissue involvement, and complex post-operative changes on MRI. Earlier detection of incomplete resection or recurrence will allow for earlier ability to intervene and potentially improve patient outcomes. Circulating-tumor DNA (ctDNA) is cell-free DNA that is released by tumor cells as they undergo cellular turn-over. Monitoring ctDNA has been shown to be more sensitive at predicting residual tumor than imaging in numerous solid malignancies. Furthermore, ctDNA could be detected earlier in peripheral blood as opposed to imaging changes, allowing for earlier intervention. In this review, we intend to give a brief overview of the current state of molecular diagnosis for skull base chordomas. We will then discuss current advances in the utilization of ctDNA for the management of CNS pathologies such as glioblastoma (GBM) and brain metastases. We will also discuss the role ctDNA has in the management of non-CNS pathologies such as osteosarcoma and Ewing sarcoma (EWS). Finally, we will discuss potential implications of ctDNA monitoring for chordoma management.

## Introduction

Chordomas are low grade tumors that reside primarily in the sacral region, skull base, or within the vertebral bodies. Known to primarily affect patients that are 40-75 years of age, this tumor accounts for approximately 3% of all bone tumors, and roughly 300 new cases of chordoma are diagnosed in the United States each year (1, 2).

Derived from remnants of the notochord, these tumors are very slow growing yet locally invasive, enabling this tumor to have a higher recurrence rate compared to other low grade CNS tumors. Specific genes that are known to contribute to the formation of chordomas include the mTOR signaling pathway, a deficiency in the PTEN pathway, and the brachyury gene, among others (3–5). Deletions of 1p36 and 9p21 (p16/CDKN2A) have been shown to be strongly predictive of outcomes and have recently been incorporated into treatment paradigms allowing for the individualization of treatment (6–8).

The centerpiece of treatment for skull base chordomas is surgical resection, however, total en bloc resection of this tumor is often challenging as it is often difficult to achieve negative margins. Surgical outcomes for this disease are difficult to determine as outcomes are often determined through intra-operative observation as well as post-operative imaging, however these methods of assessment are often inaccurate due to both the presence of unresected microscopic disease, as well as the presence of significant post-operative changes.

While patients can undergo adjuvant radiation therapy for this disease, this treatment modality is associated with significant risks (9). Studies by Abdallah and colleagues highlighted how genetic information can be helpful in determining whether skull base chordoma patients should undergo radiation therapy. Specifically, the results from this study suggested that skull base chordomas with genetic profiles that were deemed “low risk” (based on the presence of 1p36 and 9p21 deletions) could forgo radiation therapy as these patients experienced minimal benefit from this treatment which carries significant risks of complication. The phenotype for tumors with low-risk genetic profiles was defined by the authors as tumors that were smaller, less invasive, asymptomatic when discovered, and more often discovered in younger patients. While genetically profiling chordomas is helpful in determining whether to administer radiation therapy following initial surgical resection, new methods must be created to aid in determining the true extent of surgical resection and the presence of microscopic disease, as well as in determining whether patients are experiencing a recurrence so that they can be more closely monitored. ctDNA could aid in addressing these areas of concern as this technology is being used in cancers outside the CNS to monitor for tumor recurrence as well as to glean genomic information about CNS and non-CNS cancers.

Cell-free DNA (cfDNA) are DNA fragments that can be found in the blood, CSF, or other bodily fluids, that are derived from different cell populations within the body. A subtype of cfDNA is circulating-tumor DNA (ctDNA) which are DNA fragments that have been released into the blood stream from a tumor. It has been well established that tumors are often highly vascularized as tumors need an increased blood supply to continue their growth and proliferation. As tumors grow, their cells undergo apoptosis/necrosis and these cells release their DNA into the blood stream which is often adjacent to the lesion (10). This ctDNA can then be detected *via* liquid biopsy and analyzed to determine whether a tumor is recurring, as well as to establish a mutational profile for the lesion which can be exploited *via* targeted therapies.

In this review, we intend to highlight how ctDNA technology is currently being used in clinical studies to monitor for tumor recurrence in patients diagnosed with brain metastases, glioblastoma (GBM), as well as osteosarcoma and Ewing sarcoma (EWS). In addition to highlighting whether this monitoring of recurrence improved patient outcomes, we will also highlight genomic findings that were gleaned from these studies. Finally, we will share our reasons for why we believe the field should consider incorporating ctDNA monitoring into the management of patients diagnosed with skull base chordomas.

## Current molecular methods for diagnosing skull base chordomas

The current methods used for diagnosing skull base chordomas are quite limited as fine-needle aspiration or core needle biopsy are the preferred methods of diagnosis prior to surgical resection. However, these methods pose a concern for tumor seeding (11). Until recently, chordomas were diagnosed by examining their histopathological features as well as their immunoreactivity for S-100 and the presence of epithelial markers such as cytokeratins (12). However, while these methods were useful in diagnosing chordomas, it was quite difficult to differentiate between chordoma and chondrosarcoma which are managed quite differently and bear different prognoses. Recently, brachyury has been recognized as a biomarker capable of distinguishing between chordomas and other chondroid tumors. A study by Oakley and colleagues found that in tissue microarray analysis of 103 chondroid tumors residing within the skull base and other regions within the head and neck, brachyury and cytokeratin staining had a specificity and sensitivity for detecting chordomas of nearly 100% (13). Additionally, histopathologic features such as mitotic figures and a Ki-67 labeling index of greater than 6% are used as measures predictive of chordoma recurrence (14).

Given the risks associated with needle biopsy, the time is now to develop a minimally invasive molecular method for diagnosing and monitoring skull base chordomas. ctDNA analysis is a promising molecular method in the management of skull base chordomas as it has been shown that ctDNA harbors tumor specific somatic mutations, which can potentially be therapeutically exploited, and many tumors will release ctDNA into the bloodstream (15–17). Additionally, it has been shown that ctDNA can be utilized to detect microscopic disease burden up to six months before disease is able to be observed *via* conventional imaging studies (15, 16, 18). This finding highlights how blood based ctDNA monitoring may be incredibly useful in chordoma management as chordomas are often slow growing and can recur years after initial resection. Early detection of recurrence may allow for therapy to be administered earlier in a patient’s recurrence, which could lead to a potential improvement in patient outcomes.

While studies assessing the value of ctDNA analysis in chordoma management are few, one study by Mattox and colleagues found that 87.1% of patients with spinal chordomas were ctDNA positive at the time of their initial blood draw, which was prior to initial surgical resection (18). Additionally, the authors note that follow up blood draws in twenty of the thirty-two patients enrolled in this study suggested that ctDNA levels may reflect clinical status of disease however, these results were not statistically significant due to small sample size (18). Finally, the authors observed that patients who had positive ctDNA levels had greater mutant allele frequencies and were more likely to undergo radiation therapy (18). While not statistically significant, findings from this study suggest that the presence of ctDNA may be correlated with systemic response to chemotherapy and/or recurrence of disease (18).

Currently, little if any studies are present in the literature that assess whether ctDNA analysis is valuable for managing skull base chordomas. Therefore, in addition to discussing the few studies showing how ctDNA has been used in the management of chordomas, we will also discuss how this promising technology is being used in the management of brain metastases, GBM, EWS and osteosarcoma, as we hope to create a narrative for why this technology may be useful in the management of skull base chordomas.

## Brain metastases

Brain metastases are the most common neurological complications of systemic cancer, with most neurological metastases frequently found within the brain parenchyma, cranium, dura, and leptomeninges (19). Among all cancer types, lung cancer, breast cancer, and melanoma are the most frequent to metastasize to the brain (20). In many instances, patients with established diagnosis or primary malignancy don’t undergo biopsies for metastatic lesions due to the procedure’s invasiveness. Additionally, the tissue biopsy approach for brain lesion sampling is dependent upon access to the tumor, and is subject to sampling bias due to tumor heterogeneity (19). Within recent years, peripheral blood cell-free circulating tumor DNA (ctDNA) has been used to characterize and monitor various types of cancer (21, 22). Research suggests that ctDNA is not only an extremely effective method of disease assessment more so than CT imaging, but it can be used to determine response to treatment, differentiating pseudo progression from true tumor progression, and track levels of residual disease (23).

ctDNA, also referred to as liquid biopsy, carries specific gene mutations, and has been used to analyze somatic sequence alterations in various cancers through next-generation sequencing (NGS) or droplet digital polymerase chain reaction (ddPCR) (24). With the promising emergence of this biomarker, several studies have demonstrated its use in the monitoring of intracranial and extracranial brain metastases (16, 25, 26). In a large-case series that sought to determine the predictive value and limitations of peripheral blood-derived ctDNA at baseline and during treatment in patients with melanoma with brain metastases receiving PD-1 inhibitor-based therapy, ctDNA reflected extracranial melanoma activity (27). However, it was not an accurate biomarker of intracranial activity. Intracranial and extracranial disease volume was evaluated in 57 of 72 (79%) patients *via* CT and MRI at baseline. Of those evaluated, 19% had intracranial metastases only, while 81% had intracranial and concurrent extracranial metastases. ctDNA at baseline was undetectable in all patients with intracranial metastases only, while the concurrent group had a ctDNA detection rate of 70%. Intracranial disease response was evaluable in all patients, with ctDNA detectable in 53%, 0% of which were intracranial responses and 64% of which were extracranial, supporting the conclusion that ctDNA is associated with extracranial response, but not intracranial response.

Despite the either absent or extremely low levels of plasma-derived ctDNA associated with exclusive intracranial lesions, it has been shown that ctDNA is present in cerebrospinal fluid (CSF) of brain tumor patients (28). Given CSF’s intimate contact with tumor cells of the CNS derived from primary or metastatic lesions and its routine use in cytology examinations for patients with brain lesions (19), there is substantial clinical relevance in its analysis for CNS malignancies. In a recent case report published by Huang W.T. et al, ctDNA was analyzed and used to tailor the treatment of a patient with brain metastases. In a 35-year-old woman who presented with hydrocephalus due to leptomeningeal metastases and a focal mass in the spinal cord adjacent to the cerebellum, the large spinal tumor was excised with histopathological examination confirming adenocarcinoma. The patient was then given several adjuvant immunotherapeutic regimens and chemotherapy drugs, with only anti-HER2 therapy resulting in any clinical benefit. ctDNA analysis was then performed which revealed amplifications of HER2 and MPL, as well as mutations in the PIK3CA, CDKN2A and TP53 genes. Given the ctDNA mutation profile, ado-trastuzumab emtansine was added to a combination regimen of intrathecal trastuzumab and oral lapatinib. Within two weeks of the new anti-HER2 regimen combination, neurological signs increased dramatically and a significant decrease in tumor markers CEA and CA19-9 were noted. Not only does this highlight ctDNA’s ability to monitor the effectiveness of ongoing treatment, but its ability to provide insight to the tailoring of personalized treatment.

A prospective study of 21 consecutive patients with non-small cell lung cancer (NSCLC) and brain metastasis also confirmed the specificity of CSF-derived ctDNA over plasma-derived ctDNA in detecting genetic abnormalities specific to brain metastases (29). The study found that mutations were detected in the CSF ctDNA of 20 (95.2%) patients, with a detection rate of epidermal growth factor receptor (EGFR) mutations of 57.1% in CSF ctDNA versus only 23.8% in peripheral blood ctDNA and plasma circulating tumor cells (CTCs). EGFR mutations were found in the CSF of 81.8% of patients with leptomeningeal metastases, as compared with 30% of patients with brain parenchymal metastases. Additionally, the status of EGFR and TP53 mutations was consistent between CSF ctDNA and brain lesion tissue in all patients. As this study further confirms the ability of CSF ctDNA to assist in improving the management of patients with brain metastases, in terms of EGFR-driven genes, the results supported prior studies by Li et al (30) who indicated that CSF was more representative of EGR mutation status in brain metastases than plasma-derived. Additionally, its specificity gives credence to the fact that CSF ctDNA may also reveal uncommon EGFR mutations that could be targeted with specific treatments (31, 32), a consequential tool in molecular brain metastases surveillance.

Another case in a HER2-positive metastatic breast cancer patient with brain metastases also confirms CSF-derived ctDNA’s ability to harbor clinically relevant genomic alterations in patients with CNS metastases, making it an effective tool in tracking tumor evolution (33). Baseline CSF-derived ctDNA analysis revealed TP53 and PIK3CA mutations as well as ERBB2 and cMYC amplification. Post treatment ctDNA analysis showed decreased marker levels in plasma, consistent with extra-CNS disease control, while increased in the CSF, confirmed poor treatment benefit in the CNS. Consequently, these results are a positive indicator that CSF ctDNA is more suitable than plasma in revealing the mutational profile of CNS metastases, enabling the characterization of genomic complexity (23) as we seek to create optimized management and treatments for patients with known and unknown brain metastases.

## Glioblastoma (GBM)

GBM is one of the most common primary brain tumors and boasts an average life expectancy of just 15-18 months after initial diagnosis. The standard of care for GBM includes maximal surgical resection and chemoradiation therapy, however, despite these measures, patient survival remains poor. One of the major challenges associated with GBM management is monitoring for tumor recurrence as patients often undergo radiation therapy following surgical resection and it is often difficult to differentiate between tumor recurrence and radiation necrosis. This often requires patients to undergo a tumor biopsy which is highly invasive.

ctDNA has been a useful tool in the management of patients with GBM as it has enabled physicians to establish a mutational profile for one’s tumor. A study by Bettegowda and colleagues, was able to use ctDNA to detect IDH1, EGFR, TP53 and PTEN mutations in a subset of patients diagnosed with high- and low-grade gliomas (16). However, the authors note that only 10% of patients with gliomas enrolled in the study were able to have ctDNA detected whereas 100% of patients with cancer residing outside the CNS (bladder, colorectal, gastroesphogeal and ovarian) were able to have ctDNA detected. Studies performed since this have had higher ctDNA detection rates in GBM as one study by Piccioni and colleagues detected ctDNA in 50% of patients diagnosed with GBM (34). The results from this study suggest that the ability to detect ctDNA in patients diagnosed with GBM may be correlated with tumor grade and histopathology. Other studies have successfully used ctDNA to detect mutations in the TP53, EGFR, MET, PIK3CA and NOTCH1 genes, as well as the NF1, APC and PDGFRA genes in GBM (34, 35).

ctDNA monitoring has also been used to predict patient outcome in patients diagnosed with GBM that had MGMT methylation. Specifically, it was observed that patients with MGMT methylation detected *via* ctDNA analysis had an improved response and time till progression following treatment with alkylating agents (36). An additional study by Chen and colleagues found that GBM patients whose ctDNA demonstrated Alu methylation had an improved survival when compared to control (37). Studies that seek to use ctDNA as a means to monitor for GBM recurrence are few as GBMs are typically far removed from areas of the brain where CSF abounds, leaving blood as the most viable means to detect GBM ctDNA.

## Ewing sarcoma and osteosarcoma

While skull base chordomas reside within the intracranial compartment, unlike brain metastases and primary brain tumors, these bone tumors are outside of the BBB. Additionally, like other bone tumors, chordomas are considered slow growing.

Osteosarcoma is the most common primary bone tumor that primarily affects individuals between the ages of 10 and 30 years old (38). Treatment for osteosarcoma includes surgery which can be targeted to removing just the cancer or removing the affected limb (amputation) if the cancer is too widespread (39). Following surgery, patients undergo chemo-radiation therapy (39). The National Cancer Institute reports in the SEER database that if an osteosarcoma is considered “localized” the five-year survival rate is 77%. However, if this tumor has spread beyond the bone regionally or distantly, the five-year survival reduces to 65% and 26% respectively.

Ewing sarcoma (EWS) are the second most common type of primary bone cancer and are rarely found in individuals over the age of 30 (40). In contrast to treatment for osteosarcoma, patients diagnosed with EWS typically receive chemotherapy prior to surgical resection as this tumor is known to respond well to chemotherapy (41). Following chemotherapy, patients will undergo surgical resection or radiation therapy to remove any remaining disease (41). The National Cancer Institute reports in the SEER database that if the tumor is considered localized the five-year survival rate is 81%. However, if this tumor has spread beyond the bone regionally or distantly, the five-year survival reduces to 67% and 38% respectively.

ctDNA has proven to be a valuable tool in the management of patients diagnosed with osteosarcoma or EWS. In a study by Shulman and colleagues, ctDNA was detected in 53% and 57% of plasma samples from patients with newly diagnosed EWS and osteosarcoma respectively (42). In patients diagnosed with localized EWS detection of ctDNA was correlated with an inferior 3-year event free survival of 48.6% compared to a 3-year event free survival of 82.1% for those without detectable ctDNA (42). The risk of event and death increased in patients diagnosed with EWS or osteosarcoma as the levels of ctDNA increased (42). These findings are supported by Shah and colleagues whose work highlights that EWS and osteosarcoma patients with high ctDNA levels have a significantly increased risk of disease-related death and that a rise in ctDNA levels is predictive of patient relapse (43).

In a study by Krumbholz and colleagues, the authors demonstrate that ctDNA levels in treatment naïve patients are correlated with event-free and OS in patients diagnosed with EWS (44). Like Shah and colleagues, the authors observed that patients experience a reduction in ctDNA levels following treatment with chemotherapy (44). However, the authors also highlight that persistent ctDNA presence following two blocks of treatment with vincristine, ifosfamide, doxorubicin and etoposide is a strong predictor of poor survival (44).

In addition to serving as a valuable predictor of patient survival and highlighting patient response to treatment, ctDNA can reveal complex chromosomal rearrangements. In the study by Shah and colleagues, the research team was able to identify a novel EWSR1-PKNOX2 translocation in a EWS patient’s plasma at the time of their relapse (43). This translocation has not been previously described in the literature. While the impact this translocation has on patient survival is unknown, it has been well established that translocations can lead to gene fusions which have been shown to impact disease course (45). Incorporating ctDNA into the management of CNS and non-CNS cancers may allow for the detection of other translocations and gene fusions, allowing the field to develop a further understanding of how tumors progress, as well as better understand how these events impact patient outcomes.

## Discussion

In this review we have highlighted cancers of the CNS where ctDNA technology has demonstrated success in determining disease recurrence and/or allowed for the further understanding of tumor genetics (**Table 1**). It is important to note that ctDNA technology has been valuable in patient care for those diagnosed with non-CNS cancers as well (**Table 1**). In patients with breast cancer, ctDNA has become a valuable biomarker and enabled physicians to better determine overall patient prognosis (46, 47). Additionally, in patients diagnosed with non-small cell lung cancer, ctDNA has been shown to predict overall survival in patients treated with PD-L1 blockade or with chemotherapy, in addition to revealing valuable tumor genetic information (48).

**Table 1:** Clinical trials that have used ctDNA in the management of breast cancer, non-small cell lung cancer, melanoma, brain metastases, glioblastoma, osteosarcoma, EWS, and chordoma.

Trial #	Tumor Type	Detection Method Used	Collection Time	ctDNA Measurement	Trial Status
**NCT04353557**	Stage I - III Breast Cancer	liquid biopsy (blood)	1-month post-surgery	Determine ctDNA presence at first post-operative timepoint	Recruiting
**NCT04768426**	Triple-negative Breast Cancer (TNBC)	liquid biopsy (blood)	1. At initial standard adjuvant treatment 2. 6 months after standard adjuvant treatment	Characterize the ctDNA profile of TNBC in participants with residual disease after standard neoadjuvant chemotherapy receiving standard-of-care adjuvant capecitabine	Recruiting
**NCT05079074**	Late-stage Metastatic Breast Cancer	liquid biopsy (blood)	1. Before treatment 2. After two medication cycles 3. After disease progression	Determine the progression free survival ctDNA change index (12 months)	Completed
**NCT04906369**	Stage IV Breast Cancer	liquid biopsy (blood)	1. Baseline 2. 2 weeks after start of treatment 3. Beginning of each new treatment cycle	Identify patients with high ctDNA fractions in an effort to detect treatment failure (1 year)	Recruiting
**NCT03881384**	Breast Neoplasms	liquid biopsy (blood)	Prior to every chemotherapy session until first documented disease progression (up to 100 months)	Determine the concentration of ctDNA	Recruiting
**NCT05382052**	Stage IIIA Non-small Cell Lung Cancer	liquid biopsy (blood) (QIAamp Circulating Nucleic Acid Kit)	1. Before starting neoadjuvant treatment 2. At the end of the neoadjuvant treatment & before surgery 3. After surgery 4. 6 months after surgery 5. At first disease progression (if occurs within 24 months after surgery	Assess the association between the baseline ctDNA and ctDNA clearance with each one of three outcomes: after neoadjuvant treatment, before surgery and PFS	Recruiting
**NCT03465241**	Stage II-IIIA Non-small Cell Lung Cancer	liquid biopsy (blood) (Secondary Gene Sequencing -NGS)	1. Day before surgery 2. 3rd to 7th day after surgery 3. 3-4 weeks after adjuvant chemotherapy 4. 6-month intervals in following 2 years	To detect ctDNA in patients using the second generation of high-throughput gene sequencing (NGS)	Completed
**NCT04791215**	Non-Small Cell Lung Cancer	liquid biopsy (blood) (WES)	1. Baseline 2. With routine clinical blood draw	Verify radiologic response to immune checkpoint blockade (ICB) by clonal dynamics of serial ctDNA	Recruiting
**NCT04761783**	Melanoma Non-small Cell Lung Cancer	liquid biopsy (blood) (SIGNATERA)	1. Baseline 2. During routine care	To use ctDNA to determine patient PFS and OS as well as response rate and duration of response	Recruiting
**NCT03808441**	Stage III/IV Melanoma	liquid biopsy (blood)	2 to 3 months throughout study completion (an average of 1 year)	To assess decrease in ctDNA levels of mutant BRAF by >80% as an appropriate cut off for changing to immune therapy	Recruiting
**NCT05079113**	Stage III Melanoma Stage IV Cutaneous Melanoma	liquid biopsy (blood)	1. baseline 2. 3, 6, 18 months	To use ctDNA to identify genetic alterations correlating with the development of disease recurrence	Recruiting
**NCT02251314**	BRAF Mutant Melanoma	liquid biopsy (blood)	Pre-mortem - 3x 1-30 days apart (1st blood draw during 1st visit) Post-mortem - 48-72 hours from when death expected	Determine the percentage correlation between ctDNA and metastatic sites	Completed
**NCT02875652**	Choroidal Melanoma	liquid biopsy (blood)	1. Before treatment 2. 1 month after local treatment 3. 7 months 4. Every 6 months up to 3 years	Assess change of ctDNA from baseline to 3 years	Completed
**NCT02071056**	Leptomeningeal CNS Metastasis	cerebrospinal fluid	During clinically indicated procedures throughout study	To determine whether circulating tumor DNA can be identified in the CSF of patients prior to cytological evidence of leptomeningeal metastasis in patients with history of visceral cancer	Completed
**NCT04112238**	Diffuse Large B Cell Lymphoma CNS Metastasis	cerebrospinal fluid	1. At time of diagnosis 2. At time of relapse	Determine CSF tumor cfDNA and metabolite detectability and cytological/flow cytometric confirmation of CNS lymphoma at the time of diagnosis and relapse	Recruiting
**NCT05480644**	Primary Brain Tumor Brain Metastases	liquid biopsy (blood)	6 weeks post radiation therapy	To create a repository of blood samples with number of circulating immune cells and ctDNA in peripheral blood to associate with radiation therapy outcomes	Not yet recruiting
**NCT04109131**	CNS Metastases	liquid biopsy (blood) cerebrospinal fluid	Pre-diagnosis: 1. TNBC/HER2+ BC: once a year 2. NSCLC/SCLC: every 4 months 3. Melanoma: every 6 months At 1st CNS diagnosis: As close to diagnosis of CNS metastases and no later than 6 weeks after Post diagnosis: Every 3 months (+/- 1 month)	To use ctDNA to better understand the epidemiology and biology of CNS metastases derived from solid tumors	Recruiting
**NCT02060890**	Glioblastoma	liquid biopsy (blood)	1. Before surgery 2. After surgery 3. 2-month intervals (post-op)	To use ctDNA to help guide treatment plan in adults with recurrent/progressive disease	Completed
**NCT05281731**	Glioblastoma	liquid biopsy (blood)	1. 10 minutes prior to ultrasound sonication 2. 10 minutes after ultrasound sonication 3. 30 or 60 minutes after ultrasound sonication	Determine the feasibility of sonobiopsy as measured by change in ctDNA level	Recruiting
**NCT04776980**	Glioblastoma	liquid biopsy (blood)	During surgical biopsy	Determine proportion of mutations identified by biopsy that are correctly identified in samples of plasma ctDNA	Recruiting
**NCT03496402**	Osteosarcoma and Ewing Sarcoma	liquid biopsy (blood) cerebrospinal fluid	1. At time of diagnosis 2. During treatment 2. At time of relapse	Compare between genetic variations identified at diagnosis and those identified on ctDNA during treatment, FU and/or relapse	Recruiting
**NCT02736565**	Ewing Sarcoma	liquid biopsy (blood)	1. Chemotherapy Cycle 1 Week 1 Day 1 2. Chemotherapy Cycle 1 Week 3 Day 1 3. Chemotherapy Cycle 2 Week 1 Day 1 Prior to product infusion and every even cycle thereafter at week 1 day 1 prior to product infusion	Assess ctDNA (EWSR-FLI1) levels and compare to tumor burden and disease response prior to and following pbi-shRNA EWS/FLI1 Type 1 lipoplex administration	Active, not recruiting
**NCT02306161**	Ewing Sarcoma	liquid biopsy (blood)	Unknown	Determine the proportion of patients that have change in translocation result associated with ctDNA testing over different time periods	Active, not recruiting
**NCT02180867**	Osteosarcoma	liquid biopsy (blood)	At time of diagnosis	Determine the prevalence of ctDNA at the time of diagnosis	Active, not recruiting
**NCT03083678**	Chordoma	liquid biopsy (blood)	1. Baseline 2. Cycle 4 - Day 1 3. Cycle 7 - Day 1 4. End of Treatment (within 30 days of last dose)	To gather ctDNA at baseline, during treatment and at the end of treatment for patients treated with afatinib	Active, not recruiting

Few studies have utilized ctDNA in the management of chordoma, however, a study by Zuccato and colleagues revealed that chordomas have different subtypes based on their DNA methylation profiles (49). The research team found that different methylation profiles had a strong impact on clinical outcome (49). Additionally, the study found that cell-free DNA methylomes can be used to distinguish chordomas from meningiomas and spinal metastases (49).

There are many potential benefits ctDNA may provide should it be incorporated into the management of those diagnosed with chordomas. In CNS and non-CNS cancers discussed throughout this manuscript, ctDNA has been used to glean valuable tumor genetic information. Given that tumor genetics impacts chordoma invasiveness and risk of recurrence, analysis of genetic information from ctDNA may aid in determining whether patients should undergo radiation therapy, which has risks of complication (7). Additionally, genetic information gleaned from ctDNA analysis may inform patient prognosis as well as aid in understanding tumor subtype.

There are major challenges in determining chordoma recurrence *via* conventional imaging studies due to anatomical changes following surgical resection as well as the slow growing nature of chordomas (50). ctDNA has been used in determining recurrence for tumors residing outside the CNS and may be of value in determining chordoma recurrence as this tumor resides outside the BBB, allowing for the release of ctDNA into the bloodstream and subsequent detection *via* peripheral biopsy.

## Author contributions

SF performed a review of the literature, wrote the manuscript, and is the first author. CD contributed to the writing of the manuscript and created the table included in the manuscript. XZ, SH, SA, PG, CS, EW, and GZ provided their expertise on the clinical aspects of the manuscript. GZ supervised the writing of the manuscript. All authors contributed to the article and approved the submitted version.

## Conflict of interest

The authors declare that the research was conducted in the absence of any commercial or financial relationships that could be construed as a potential conflict of interest.

## Publisher’s note

All claims expressed in this article are solely those of the authors and do not necessarily represent those of their affiliated organizations, or those of the publisher, the editors and the reviewers. Any product that may be evaluated in this article, or claim that may be made by its manufacturer, is not guaranteed or endorsed by the publisher.
